# The synovial fluid fibroblast-like synoviocyte: A long-neglected piece in the puzzle of rheumatoid arthritis pathogenesis

**DOI:** 10.3389/fimmu.2022.942417

**Published:** 2022-08-05

**Authors:** Dorra Elhaj Mahmoud, Wajih Kaabachi, Nadia Sassi, Lamjed Tarhouni, Sonia Rekik, Samia Jemmali, Hela Sehli, Maryam Kallel-Sellami, Elhem Cheour, Lilia Laadhar

**Affiliations:** ^1^ Immuno-Rheumatology Research Laboratory, Rheumatology Department, La Rabta Hospital, University of Tunis-El Manar, Tunis, Tunisia; ^2^ Unité de Recherche Infections Respiratoires Fongiques (IRF), Structure Fédérative de Recherche “Interactions Cellulaires et Applications Thérapeutiques” (SFR ICAT), Université d’Angers, Angers, France; ^3^ Department of Hand and Reconstructive Surgery, Kassab Institute of Traumatic and Orthopedic Surgery, Tunis, Tunisia

**Keywords:** rheumatoid arthritis, synovial fluid, fibroblast-like synoviocyte, inflammation, PRIME cells, fibrocyte, joint destruction

## Abstract

Rheumatoid arthritis (RA) is a systemic autoimmune disease during which fibroblast-like synoviocytes (FLS) contribute to both joint inflammation and destruction. FLS represent the core component of the synovial membrane. Following inflammation of this membrane, an effusion of cell-rich synovial fluid (SF) fills the joint cavity. Unlikely, SF has been shown to contain fibroblasts with some shared phenotypic traits with the synovial membrane FLS. These cells are called SF-FLS and their origin is still unclear. They are either brought into the synovium *via* migration through blood vessels, or they could originate within the synovium and exist in projections of the synovial membrane. SF-FLS function and phenotype are poorly documented compared to recently well-characterized synovial membrane FLS subsets. Furthermore, no study has yet reported a SF-FLS single-cell profiling analysis. This review will discuss the origin and cellular characteristics of SF-FLS in patients with RA. In addition, recent advances on the involvement of SF-FLS in the pathogenesis of RA will be summarized. Current knowledge on possible relationships between SF-FLS and other types of fibroblasts, including synovial membrane FLS, circulating fibrocytes, and pre- inflammatory mesenchymal (PRIME) cells will also be addressed. Finally, recent therapeutic strategies employed to specifically target SF-FLS in RA will be discussed.

## Introduction

Rheumatoid arthritis (RA) is a chronic autoimmune disease that affects approximately 1% of the world’s population (1). The etiology of RA has not yet been fully understood and genetic, environmental and stochastic factors have been contended to be involved (2). It has been reported that a combination of epigenetic modifications and environmental factors can lead to modified self-antigens such as immunoglobulin G (IgG), collagen II, fibrin and vimentin. These proteins with arginine residues can be converted to citrulline by peptidyl arginine deiminases, and this post-translational modification is called citrullination. During RA, an autoimmune response is developed against citrullinated peptides detected as anti-citrullinated peptide antibodies (ACPA) (3). RA is also characterized by persistent inflammation, frequently resulting in bone erosion and joint destruction if left untreated (4). An effusion of synovial fluid (SF), which fills the joint cavity, is also observed. SF is known to contain several immune cells and inflammatory cytokines, together entertaining inflammation in the affected area (5). RA pathology involves abnormalities of both the immune system (including T cells, B cells, plasma cells, dendritic cells, macrophages and mast cells) and fibroblast-like synoviocyte (FLS) activity (6, 7).

FLS, also known as type B synoviocytes, have been identified as important cellular players in RA pathogenesis (8). There is growing evidence that FLS activation is an early step in the development of RA (6). Indeed, RA FLS exhibit an aggressive phenotype and produce pathogenic inflammatory mediators such as cytokines. They also produce matrix-degrading enzymes that promote local inflammation and disease perpetuation. Furthermore, RA FLS are involved in antigen presentation to autoreactive T cells (1, 9–11). Their excessive proliferation and resistance to apoptosis are the cause of synovial hypertrophy. Their migratory and invasive properties exacerbate joint damage (12). Interestingly, different FLS subsets with distinct functions have been characterized in the synovial tissue (13–15). In addition to tissue-resident cells, FLS have also been found in the synovial fluid (SF) of patients with RA (16–19).

There is an increasing interest within the research community in analyzing tissue-resident FLS and their role in the development of RA. Recent data confirm the importance of these cells in synovial inflammation, joint destruction and even disease spread into distal sites (2, 7, 8).

This mini review will cover recent insights about the cellular characteristics and involvement of SF-FLS in the RA pathogenesis. The eventual relationship between RA SF-FLS and the other fibroblast populations, including tissue-resident FLS, fibrocytes and pre-inflammatory mesenchymal (PRIME) cells will be focused on. Finally, recent therapeutic strategies employed to specifically target SF-FLS in RA will be discussed.

## Origin of SF-FLS

The exact origin of SF-FLS has not yet been determined. However, there are different opinions on the possible source of these cells (16–20). The first one suggests that SF-FLS arise from circulating progenitors introduced into the synovium in response to cytokines produced in the inflamed joint (17). This hypothesis was proposed after the identification of circulating cells with fibroblastic characteristics in the peripheral blood of patients with RA (21–23).

Circulating human fibrocytes, which have already been shown to differentiate into mature fibroblasts, are the best candidates to be the origin of SF-FLS (22).

Fibrocytes are circulating progenitor cells of the mesenchymal lineage and represent approximately 0.5% of peripheral blood leukocytes (24, 25). They express CD34 and produce components of the connective tissue matrix, including collagen I (col I) and fibronectin (26, 27). Fibrocytes are among the earliest responding cells in the innate response to injury or tissue invasion (21, 28). Galligan et al. provided evidence that circulating fibrocyte cells migrate into the joints during the inflammatory process and that an adoptive transfer of activated fibrocytes in recipient mice enhances the disease process (29). In addition, high percentages of fibrocytes have been found in both blood and SF of patients with RA. Interestingly, SF-fibrocytes have low CD34 expression which reflects an eventual cell differentiation (30). While SF-FLS reappear in joints after synovectomy indicating possible migration and differentiation of progenitors into FLS (17), the possibility that these cells may emerge from the synovial membrane after desquamation of lining or sub-lining FLS cannot be excluded (31).

## Culture of SF-FLS

SF-FLS can be isolated from synovial fluid and grown in culture for several prolonged passages. SF is generally aspirated from the knee, wrist or elbow during an RA flare-up (16–19, 31). Centrifugation of SF gives a cell pellet, and some cells are able to adhere to tissue culture dishes. Non-adherent cells are eliminated, leaving a mixture of two major cell populations: SF-FLS and SF-macrophages (16, 17). SF-macrophages are terminally differentiated cells with a limited lifespan *in vitro*, and rarely survive more than a few weeks in culture. SF-FLS proliferate rapidly in passages 1 through 4, generally reaching confluence within 10 to 20 days, but grow more slowly in later passages (16–19, 31, 32). Several cultures were maintained for over one year, reaching passage 9 or 10 (16, 17). The culture of SF-FLS is considerably longer than that of synovium FLS (33). The prevalence of positive cultures varies from a study to another. Generally, SF-FLS are obtained from 60% to 80% of SF from RA patients during the disease flare-up (16, 18, 19). Neidhart et al. demonstrated that the presence of RA FLS in SF can be affected by many clinical factors (16). However, Stebulis et al. found that patient’s treatment, initial fluid volume, or cell counts are not associated with the presence of RA-FLS in SF (17).

## Morphology of cultured SF-FLS

SF-FLS from patients with RA display multiple changes in cell morphology during culture (17, 18, 34). Under light microscopy, SF-FLS appear elongated with an irregular stellate cell shape during the first few days of culture (16, 17). In primary cultures, SF-FLS are frequently observed close to cells with macrophage morphology (17). Less frequently, SF-FLS start proliferating only after separation from macrophages (16). After the first weeks of culture, SF-FLS became larger and actively proliferative. In most cultures, spindle-shaped cells appear to develop from dense cellular clusters, but isolated cells are also observed (17). Similar to the changes observed in FLS isolated from RA synovium, SF-FLS from RA patients grow in an anchorage-independent mode and form villous projections that float freely in the culture medium. Furthermore, SF-FLS exhibit the ability to form a tissue-like structure in culture (17, 18). The tissue border is composed of multiple layers of SF-FLS aligned longitudinally. Non-RA cultures of SF-FLS are unable to form any tissue-like structures (17).

## SF-FLS *versus* other fibroblasts/mesenchymal stem cells

Recent studies support the idea that FLS constitute a heterogeneous population and that distinct subtypes of FLS do exist. These cells are involved differently in the pathogenesis of RA. They differ in their gene expression patterns, cellular markers and epigenetic signature (14, 15, 33). The synovium also houses mesenchymal stem cells (MSC). While both SF-FLS and MSC are part of the synovium their connection remains unclear. Possible relationships between SF-FLS and the other fibroblasts/MSC will be discussed below and summarized in **Table 1**.

**Table 1:** Profile of SF-FLS surface markers.

SF-FLS marker	Other fibroblast/MSC in which it is found	Functions	References
**CD55**	Lining FLS	Oncogenic properties such as invasion and migration	**(** 16, 35, 36 **)**
**PDPN**	Sub-lining FLS PRIME cells	Mediates tumor cell migration and invasion Play a role in severe RA	**(** 19, 23, 35 **)**
**THY1 (CD90)**	Sub-lining FLS SF-MSC	Involved in tissue destruction by highly expressing genes related to osteoclast differentiation or activation, such as receptor activator of nuclear factor-κB ligand (RANKL)	**(** 15, 19, 35 **)**
**HLA-DR**	Fibrocytes Sub-lining FLS	Antigen presenting molecule	**(** 19, 37 **)**
**CDH11**	Lining FLS PRIME cells	Critical for homotypic aggregation of FLS *in vitro* and *in vivo*	**(** 20, 23, 27 **)**
**CXCR3**	Fibrocytes	Mediates cell invasion	**(** 27, 38 **)**
**IL1RI**	Fibrocytes	Involved in IL1 induced inflammatory responses	**(** 27 **)**
**Wnt receptors : Fzd4, Fzd5, Ror2, Ryk, LRP5**	Fibrocytes	Mediate Wnt signaling Involved in cell migration and Inflammation	**(** 27 **)**
**ICAM1**	Sub-lining FLS	Expressed by FLS and linked to their inflammatory properties	**(** 19 **)**

### SF-FLS versus lining and sub-lining FLS

The term synovium refers to a thin membrane that encapsulates the joint cavity (39). This membrane is divided into two regions: the intima or synovial lining composed of intimal lining FLS and macrophages, and the sub-lining layer or sub-intima that contains principally sub-lining FLS, macrophages and adipose cells (40, 41). During RA, FLS hyperplasia occurs in both locations (42).

Tissue-resident FLS have long been considered as functionally homogeneous cells. However, it is now widely accepted that these cells are heterogeneous groups that perform a number of distinct and specialized functions (13).

Recently, Stephenson et al. reported two distinct human FLS subsets in RA synovium using single cell RNA sequencing (scRNA seq) transcriptomics. The first subset is localized in the intima and expresses complement decay-accelerating factor or CD55 molecule (*CD55^+^* lining FLS) while the second subset is found in the sub-intima and expresses thymocyte differentiation antigen1 (THY1) or CD90 marker (THY1+ sub-lining FLS) (35).

Lately, Zhang et al. described some heterogeneity in the sub-lining layer FLS and defined three THY1+ groups with additional subset markers: CD34 specific to the first group, human leukocyte antigen (HLA)-DRA-high that characterized the second group and Dickkopf 3 (DKK3)that defined the third group (43) (43).

In order to phenotypically characterize the SF-FLS of patients with RA, Koster et al. used three surface markers: podoplanin (PDPN), THY1 and CD34 (19). After a few passages, the SF-FLS cultures consist of primarily PDPN+ THY1^+^CD34^−^ cells. Furthermore, following co-cultures with immune cells or INFγ, SF-FLS express HLA-DR.

These results demonstrate that SF-FLS from patients with RA share phenotypic characteristics with a pathogenic subset of sub-lining RA-FLS (19). However, Neidhart et al. showed by flow cytometry the presence of common markers between SF-FLS and intimal lining FLS during RA including the intimal lining marker CD55 (16).

### SF-FLS versus SF-mesenchymal stem cells

MSC were initially isolated from bone marrow (44). *In vitro*, they can adhere to plastic culture-dishes and undergo differentiation into mature mesenchymal cells especially osteoblasts, chondrocytes and adipocytes (44, 45).

Moreover, MSC express the surface markers CD73, THY1, and CD105. They are negative for CD45, CD34, CD14, CD11b, CD79α, CD19, and HLA-DR surface molecules (46).

De Bari et al. characterized multipotent MSC from adult human synovium (47). In this context, it has been shown that MSC can be isolated from the synovial fluid of patients with RA (48, 49). The precise role of MSC in RA pathology remains unclear. In experimental arthritis, joint inflammation is preceded by infiltration of MSC, which may contribute to synovial membrane hyperplasia (50). SF-MSC are obtained *in vitro* according to the same protocol used for the SF-FLS culture. After SF culture, SF-MSC are maintained in culture with the plastic-adherent mononuclear cell fraction and can be cultured as fibroblast-like cells (48). Meanwhile, the relationship between the SF-MSC and SF-FLS remains unclear (39).

It has been proven that SF-FLS express several adhesion molecules, such as the InterCellular Adhesion Molecule 1 (ICAM1) and fibroblast makers, such as collagen and vimentin (19, 20, 27). Similarly, SF-MSC are reported to express the majority of these markers (37).

### SF-FLS versus pre- inflammatory mesenchymal cells

Recently, Orange et al. reported the presence of circulating fibroblast-like cells in the blood of patients with RA a few weeks before the disease flare-up (23). These cells are identified as PRe-inflammatory MEsenchymal (PRIME) cells. PRIME cells share some markers with SF-FLS such as cadherin 11(CDH11) and HLA-DR. Furthermore, these cells display cellular characteristics of certain sub-lining FLS subsets (23).

### SF-FLS versus circulating fibrocytes

During RA, the increased number of FLS in the synovium contrasts with the relatively low number of mitotic cells (51). This observation led Galligan et al. to study the involvement of circulating fibrocytes in the pathogenesis of RA. They provided evidence that circulating fibrocyte cells migrate into the joints during the inflammatory process (22). They also hypothesized that circulating fibrocytes may be precursors of FLS.

A recent work by Elhaj Mahmoud et al. showed that SF-FLS and fibrocytes from patients with RA express the same levels of IL1receptor I (IL1RI). The expression of IL8 receptors (CXCR1 and CXCR2) is restricted to fibrocytes while the expression of CXCR3 is reported in both cell types (27).

This chemokine receptor and its ligands CXCL9, CXCL10 and CXCL11 are involved in various inflammatory diseases, such as RA, multiple sclerosis, transplantation rejection, atherosclerosis, and inflammatory skin diseases (38).

Additionally, SF-FLS and fibrocytes from patients with RA express the Wingless (Wnt) receptors: Frizzled (Fzd) 4, Fzd5, Ror2, Ryk and the low density lipoprotein receptor-related protein 5 (LRP5). These receptors play an important role in FLS-mediated inflammatory response and cell migration (34).

## Involvement of SF-FLS in the pathogenesis of RA

### The transformed phenotype of RA SF-FLS

RA FLS display certain unique features of transformed cells. Once activated, RA FLS have their phenotype significantly modified. They exhibit the characteristics of transformed cells, including loss of contact inhibition (52, 53). Indeed, it has been demonstrated that SF-FLS proliferation in culture continued despite the formation of a confluent cell layer (17). This characteristic was initially described in the cultured lining and sub-lining FLS from patients with RA (54).

The transformation of SF-FLS phenotype during RA suggests specific alterations in the transcription and the epigenetic profile of disease-relevant genes as well as intracellular signaling pathways, including alterations in apoptotic cascades (52–54).

These changes comprise upregulation of several proto-oncogenes as well as downregulation of potentially protective tumor suppressor genes (52–54). Numerous oncogenes involved in cell cycle regulation or acting as transcription factors such as c-Fos, Cyclin E and Wnt1-inducible-signaling pathway protein 1 (WISP1) are expressed at high levels in RA SF-FLS (18).

Epigenetic modifications in SF-FLS during RA are also important. Gene-specific methylation changes are detected in RA SF-FLS (55). Glossop et al. identified a total of 195 genes that display statistically significant methylation changes in the SF-FLS from patients with RA. A hypomethylation of multiple genes has been described, such as A Disintegrin And Metalloproteinase with Thrombospondin Motifs 14 (ADAMTS14), a member of the ADAMTS family of proteinases and Methionine Sulfoxide Reductase A (MSRA), an enzyme involved in the response to oxidative damage (55). SF-FLS from patients with RA present a hypomethylation of MIR155HG, a host gene of the miRNA miR-155, involved in arthritis and cancer (55, 56). This aberrant methylation change of MIR155HG is observed in SF-FLS but not in synovium FLS (55). In addition, SF-FLS express an adhesion molecule called CDH11 crucial for the establishment of synovial architecture (20, 27). CDH11 promotes FLS migration and invasion. Furthermore, this protein enhances the production of matrix metalloproteinases (MMP) and cytokines by RA FLS (57–59). SF-FLS express fibronectin which enhances cell survival and facilitates cell adhesion to the surface of cartilage. Fibronectin is also a potent chemoattractant for fibroblasts and may facilitate chemokine signaling (18, 60, 61).

### SF-FLS and inflammation

**Figure 1:**
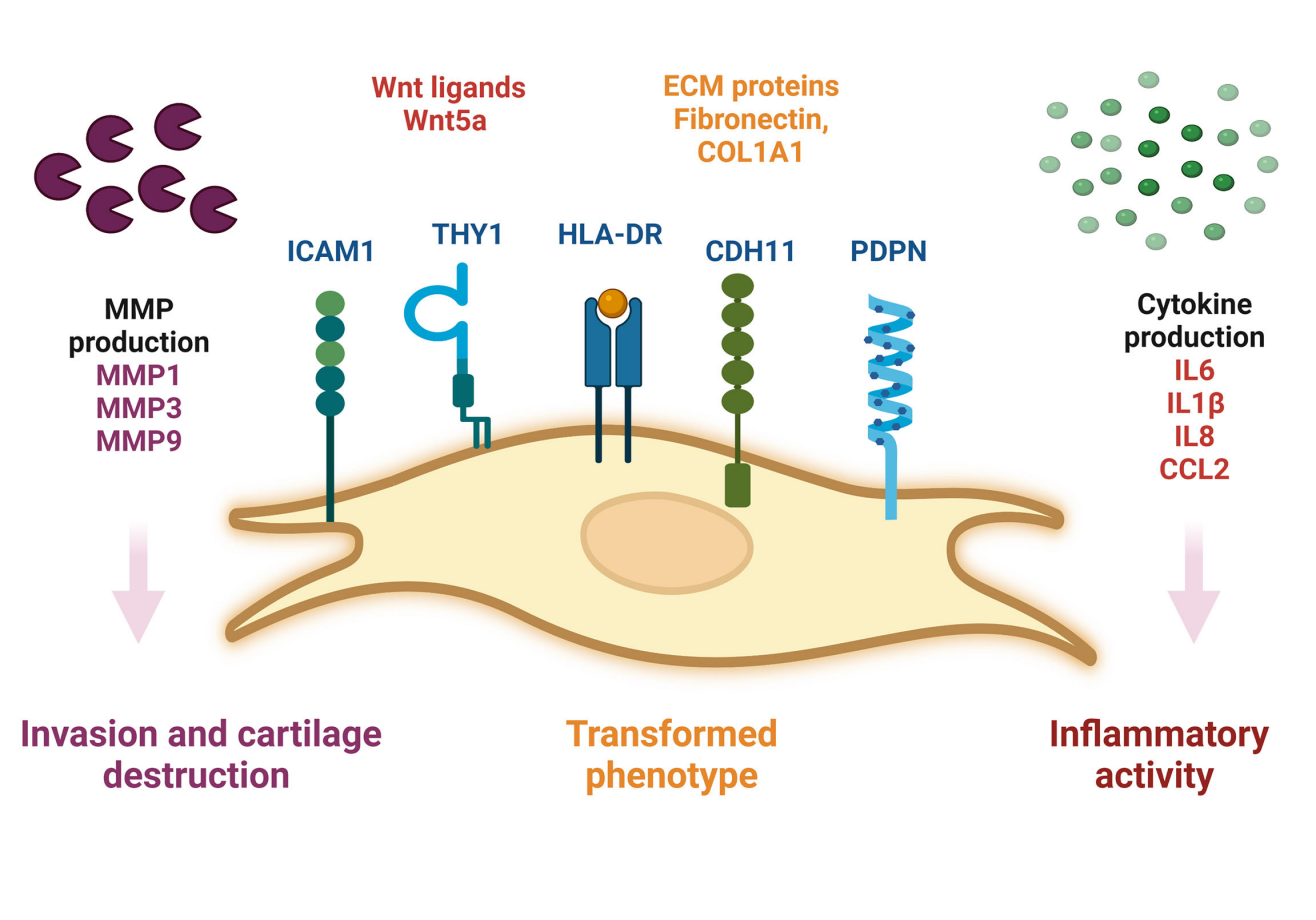
Schematic representation of SF-FLS from patients with RA. SF-FLS express THY1, HLA-DR, PDPN, ICAM1 and CDH11. SF-FLS may contribute to RA pathogenesis, notably through cytokine production and MMP secretion. SF-FLS, Synovial Fluid-Fibroblast-Like Synoviocytes; THY1, THYmocyte differentiation antigen 1; HLA-DR, MHC class II cell surface receptor; PDPN, Podoplanin; ICAM1, InterCellular Adhesion Molecule 1, CDH11, cadherin 11, MMP: Matrix Metalloproteinases.

During RA, SF-FLS actively participate in synovial inflammation. It has been shown that RA SF-FLS release multiple inflammatory cytokines dominated by CCL2 and IL6 without exogenous stimulation (19). This corroborates the findings of previous data showing that RA SF-FLS secrete IL6, IL8, IL1β and CCL2 (17, 34). In addition, RA FLS from the intimal lining have been considered primary sources of IL6, as shown by *in situ* hybridization and immunohistochemistry studies (6). However, recent data showed that SF-FLS express higher levels of IL6 than tissue FLS and fibrocytes from patients with RA (34). The release of these cytokines contributes to joint inflammation and cell recruitment into the inflamed synovium (1, 6). The inflammatory profile of RA SF-FLS reflects the activation of numerous signaling systems, including the Wnt pathway (62). Functionally, aberrant expression of Wnt signaling components has been observed in RA FLS, including SF-FLS (34, 62). Wnt5a is particularly overexpressed in RA SF-FLS (62). This Wnt ligand is involved in synovial inflammation and induces the production of numerous pro-inflammatory cytokines (62, 63) (**Figure 1**)

### RA SF-FLS and cartilage invasion/destruction

One of the first described and most characteristic features of RA SF-FLS is their ability to invade and destroy cartilage (16). It has been demonstrated that RA SF-FLS mediate cartilage degradation independently of the hyperplastic synovial tissue (16, 19). This potential is attributed to a combination of adhesion-facilitating factors and production of proteases especially the well-known MMP (16, 20).

MMP are a family of proteinases that degrade and remodel multiple components of the extracellular matrix (ECM), including collagens, fibronectin and hyaluronan (64, 65). Their enzymatic activity controls significant cellular functions such as proliferation, adhesion and migration (66, 67). MMP3 plays a pivotal role in cartilage destruction (68, 69). Previous studies have shown that SF-FLS produce a high level of MMP3 (20, 27). During RA, baseline levels of MMP3 serve as a biomarker of progressive cartilage damage. MMP3 itself may be responsible for the activation of other MMP resulting in the degradation of ECM proteins in cartilage (68). RA SF-FLS secrete MMP1 and MMP9, which are also involved in cartilage destruction (16, 20, 27). Adhesion molecules including, ICAM1 and Vascular Cell Adhesion Molecule1 (VCAM-1), facilitate the anchoring of RA FLS to the cartilaginous ECM components (6). A recent study showed that ICAM1 is highly expressed in cultured SF-FLS from patients with RA (19). Furthermore, CDH11, which is expressed by SF-FLS during RA, is relevant in cartilage destruction (6).

### RA SF-FLS as potential migratory cells

Migration and invasion are complex processes that require dynamic interactions between cells and the surrounding matrix.

Fibroblast migration in human disease has only recently been reported (13). The first cell with fibroblastic proprieties described to migrate from the circulation into joint was fibrocytes (70). The discovery of detectable pre-inflammatory PRIME cells in the blood of patients prior to an arthritis flare-up has revived the debate on synovial fibroblasts migration (13, 23).

**Figure 2:**
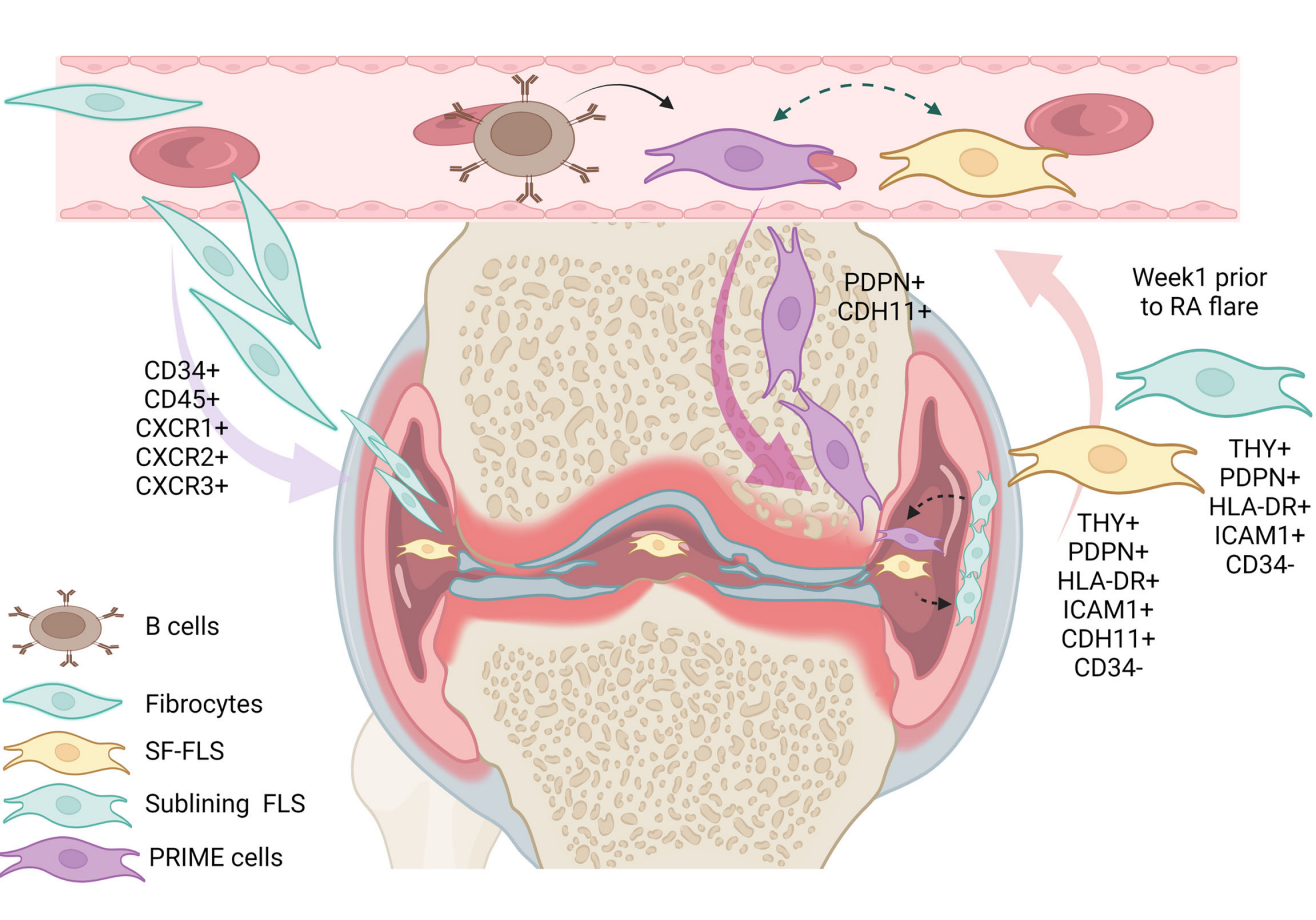
Illustration of the eventual relation between SF-FLS, fibrocytes, sub-lining FLS and PRIME cells. It has been shown that fibrocytes from patients with RA have a migratory potential with the capacity to leave the blood and reach the inflamed joint. Within the inflamed joint, fibrocytes differentiate into FLS. According to this model, SF-FLS may constitute a fibrocyte differentiation state. New data confirm that SF-FLS have a sub-lining layer phenotype. PRIME cells are also thought to have a phenotype similar to both SF-FLS and a sub-lining FLS subset. PRIME cells are activated by B cells and their number increases in the blood prior to a flare-up and migrate into the joint to enhance inflammation. SF-FLS, Synovial-Fluid Fibroblast-Like Synoviocytes; PRIME cells, PRe-Inflammatory MEsenchymal cells.

Previously, some authors supposed that SF-FLS have the ability to migrate and “metastasize” from one joint to another, and potentially spread the disease (16, 19). However, this idea remains unproven. Interestingly, SF-FLS and a subset of sub-lining FLS share proprieties with recently described PRIME cells (19, 23). PRIME cells are thought to have a sub-lining layer phenotype. The authors proposed that peripheral blood B cells lead to the activation of PRIME cells in the circulation and the migration of these cells into the synovium (23).The question remains: what is the relation between SF-FLS, sub-lining FLS and PRIME cells? It is tempting to speculate that SF-FLS, sub-lining FLS and PRIME cells are the same cell type. SF-FLS may derive from the sub-intimal lining and fall into the SF. These cells may migrate through the circulation, increase in blood before a flare-up and then decrease just after symptom onset (**Figure 2**).

## Targeting SF-FLS in RA

Despite great advances in RA therapy, numerous patients with RA have persistent disease. Current daytime treatment strategies for RA focus on suppressing cytokine signaling and T- and B-cell activity (1).

Certain molecules including Janus Kinase (JAK) inhibitors have been shown to reduce FLS aggressiveness. However, therapies that selectively target FLS are still lacking (71).

FLS-targeted therapies present a nice advantage as they could potentially be used in combination with immune suppressors with limited side effects on host defense. So far, only CDH11 has emerged as a potential target for selective anti-FLS therapy. Indeed, CDH11 is expressed by SF-FLS during RA (27) and is involved in RA pathogenesis. However, a phase II study of a monoclonal antibody directed against CDH11 (RG6125) was discontinued in 2019 due to a lack of efficacy (72).

Several observations have suggested that targeting the TNF-related apoptosis inducing ligand (TRAIL), a member of the TNF family or its death receptors TRAIL-R1 (DR4) and TRAIL-R2 (DR5) may have a therapeutic role in RA. A previous study by Miranda‐Carús et al. demonstrated that SF-FLS from patients with RA express DR5 and undergo apoptosis when exposed to an agonistic anti-DR5 antibody (31).

Recently, Notch3, a transmembrane receptor of the Notch family, has been described as a new therapeutic FLS target. Indeed, Wei et al. showed that the injection of Notch3-neutralizing monoclonal antibody attenuates the severity of arthritis in mouse model. This receptor is principally expressed by THY1+ FLS and transduces signals from endothelial cells to FLS to expand during RA (73). To the best of our knowledge, no study has yet reported Notch3 expression in SF-FLS.

## Conclusion

The heterogeneity of FLS subsets strengthens their pathogenic role in the pathogenesis of RA. Among the different FLS subsets, SF-FLS remain poorly studied and characterized. These synovial fluid-derived cells exhibit the same cellular properties as those of transformed cells, including loss of contact inhibition when cultured. During RA, SF-FLS secrete pro-inflammatory cytokines and MMP and actively participate in cartilage destruction. Phenotypically, SF-FLS share cellular characteristics with a pathological sub-lining FLS subset. SF-FLS also share properties with the recently described PRIME cells, found in increased numbers in patients with RA preceding disease flares. A thorough characterization of SF-FLS will not only help to identify new therapeutic targets, but also to understand the pathogenesis of a disease that remains a major public health problem.

## Author contributions

DM, conceived the study, wrote the manuscript and prepared the figures. WK and LL, wrote the manuscript and prepared the table. NS, LT, SR, SJ, HS, MK-S, EC contributed to the layout organization and writing. All authors approved the final manuscript

## Funding

This work was supported by funding from the Tunisian Ministry of High Education, Research and Technology.

## Acknowledgments

The authors sincerely acknowledge Dr. Mohamed Jemaa (Department of Laboratory Medicine, Translational Cancer Research, Faculty of Medicine, Lund University, 22381, Lund, Sweden) and Faten Tlili for critical reading of the manuscript.

## Conflict of interest

The authors declare that the research was conducted in the absence of any commercial or financial relationships that could be construed as a potential conflict of interest.

## Publisher’s note

All claims expressed in this article are solely those of the authors and do not necessarily represent those of their affiliated organizations, or those of the publisher, the editors and the reviewers. Any product that may be evaluated in this article, or claim that may be made by its manufacturer, is not guaranteed or endorsed by the publisher.

## Abbreviations

ACPA, Anti-Citrullinated Peptide Antibodies; CD55, Complement Decay-accelerating Factor; CDH, Cadherin; DKK, Dickkopf; ECM, Extracellular Matrix; FLS, Fibroblast-Like Synoviocytes, Fzd, Frizzled; HLA, Human Leukocyte Antigen; ICAM1, Intercellular Adhesion Molecule 1; IgG, Immunoglobulin G; JAK, Janus Kinase; LRP5, Low density lipoprotein Receptor-related Protein5; MMP, Matrix Metalloproteinases; MSC, Mesenchymal Stem Cells; PRIME cells, PRe-Inflammatory MEsenchymal cells; RA, Rheumatoid Arthritis; scRNA-seq, Single cell RNA sequencing; SF, Synovial Fluid; THY1, THYmocyte differentiation antigen-1; TRAIL, TNF-Related Apoptosis Inducing Ligand; VCAM1, Vascular Cell Adhesion Molecule 1; Wnt, Wingless.

## References

[1] Nygaard G Firestein GS. Restoring synovial homeostasis in rheumatoid arthritis by targeting fibroblast-like synoviocytes. Nat Rev Rheumatol (2020) 16(6):316–33. doi: 10.1038/s41584-020-0413-5 PMC7987137 32393826

[2] Lomholt S Nielsen MA Aspari MP Jørgensen PB Croft AP Buckley C. Fibroblast like synovial cell subsets in rheumatoid arthritis In:Bertoncelj MF Lakota K. editors Fibroblasts: Adv Inflammation Autoimmun Cancer IntechOpen(2021). doi: 10.5772/intechopen.99240

[3] Radu AF Bungau SG. Management of rheumatoid arthritis: An overview. Cells (2021) 11:2857. doi: 10.3390/cells10112857 PMC8616326 34831081

[4] Shams S Martinez JM Dawson JR Flores J Gabriel M Garcia G. The therapeutic landscape of rheumatoid arthritis: Current state and future directions. Front Pharmacol (2021) 12:680043. eCollection 2021. doi: 10.3389/fphar.2021.680043 34122106 PMC8194305

[5] Oliviero F Galozzi P Ramonda R de Oliveira FL Schiavon F Scanu A. Unusual findings in synovial fluid analysis: A review. Ann Clin Lab Sci (2017) 47(3):253–9. 28667024

[6] Bartok B Firestein GS. Fibroblast-like synoviocytes: Key effector cells in rheumatoid arthritis. Immunol Rev (2010) 233(1):233–55. doi: 10.1111/j.0105-2896.2009.00859.x PMC2913689 20193003

[7] Chen Y Dang J Lin X Wang M Liu Y Chen J. Ra Fibroblast-like synoviocytes derived extracellular vesicles promote angiogenesis by mirna-1972 targeting P53/Mtor signaling in vascular endotheliocyte. Front Immunol (2022) 13:793855. doi: 10.3389/fimmu.2022.793855 35350778 PMC8957937

[8] Li G Xia Z Liu Y Meng F Wu X Fang Y. Sirt1 inhibits rheumatoid arthritis fibroblast-like synoviocyte aggressiveness and inflammatory response via suppressing nf-κb pathway. Biosci Rep (2018) 38(3). doi: 10.1042/bsr20180541 PMC6013706 29784872

[9] Yoshitomi H. Regulation of immune responses and chronic inflammation by fibroblast-like synoviocytes. Front Immunol (2019) 10:1395. doi: 10.3389/fimmu.2019.01395 31275325 PMC6593115

[10] Ueno A Yamamura M Iwahashi M Okamoto A Aita T Ogawa N. The production of Cxcr3-agonistic chemokines by synovial fibroblasts from patients with rheumatoid arthritis. Rheumatol Int (2005) 25(5):361–7. doi: 10.1007/s00296-004-0449-x 15004722

[11] Tran CN Davis MJ Tesmer LA Endres JL Motyl CD Smuda C. Presentation of arthritogenic peptide to antigen-specific T cells by fibroblast-like synoviocytes. Arthritis Rheum (2007) 56(5):1497–506. doi: 10.1002/art.22573 17469112

[12] Benedetti G Bonaventura P Lavocat F Miossec P. Il-17a and tnf-α increase the expression of the antiapoptotic adhesion molecule amigo-2 in arthritis synoviocytes. Front Immunol (2016) 7:254. doi: 10.3389/fimmu.2016.00254 27446084 PMC4922130

[13] Marsh LJ Kemble S Reis Nisa P Singh R Croft AP. Fibroblast pathology in inflammatory joint disease. Immunol Rev (2021) 302(1):163–83. doi: 10.1111/imr.12986 34096076

[14] Micheroli R Elhai M Edalat S Frank-Bertoncelj M Bürki K Ciurea A. Role of synovial fibroblast subsets across synovial pathotypes in rheumatoid arthritis: A deconvolution analysis. RMD Open (2022) 8(1):e001949. doi: 10.1136/rmdopen-2021-001949 34987094 PMC8734041

[15] Mizoguchi F Slowikowski K. Functionally distinct disease-associated fibroblast subsets in rheumatoid arthritis. Nat Commun (2018) 9(1):789. doi: 10.1038/s41467-018-02892-y 29476097 PMC5824882

[16] Neidhart M Seemayer CA Hummel KM Michel BA Gay RE Gay S. Functional characterization of adherent synovial fluid cells in rheumatoid arthritis: Destructive potential in vitro and in vivo. Arthritis Rheum (2003) 48(7):1873–80. 10.1002/art.11166 12847681

[17] Stebulis JA Rossetti RG Atez FJ Zurier RB. Fibroblast-like synovial cells derived from synovial fluid. J Rheumatol (2005) 32(2):301–6. 15693092

[18] Elhaj Mahmoud D Sassi N Drissi G Barsaoui M Zitouna K Sahli H. Sfrp3 and Dkk1 regulate fibroblast-like synoviocytes markers and wnt elements expression depending on cellular context. Immunol Invest (2017) 46(3):314–28. doi: 10.1080/08820139.2016.1267204 28151034

[19] Køster D Egedal JH Lomholt S Hvid M Jakobsen MR Müller-Ladner U. Phenotypic and functional characterization of synovial fluid-derived fibroblast-like synoviocytes in rheumatoid arthritis. Sci Rep (2021) 11(1):1–11. doi: 10.1038/s41598-021-01692-7 34772990 PMC8590001

[20] Ahn JK Kim H Lee J Bae E-K Cha H-S Koh E-M. Phenotypic characterization and invasive properties of synovial fluid-derived adherent cells in rheumatoid arthritis. Inflammation (2008) 31(6):365–71. doi: 10.1007/s10753-008-9087-x 18850260

[21] Bucala R Spiegel LA Chesney J Hogan M Cerami A. Circulating fibrocytes define a new leukocyte subpopulation that mediates tissue repair. Mol Med (1994) 1(1):71–81. 8790603 PMC2229929

[22] Galligan CL Fish EN. The role of circulating fibrocytes in inflammation and autoimmunity. J Leukoc Biol (2013) 93(1):45–50. doi: 10.1189/jlb.0712365 22993208

[23] Orange DE Yao V Sawicka K Fak J Frank MO Parveen S. Rna identification of prime cells predicting rheumatoid arthritis flares. N Engl J Med (2020) 383(3):218–28. doi: 10.1056/NEJMoa2004114 PMC7546156 32668112

[24] Chong SG Sato S Kolb M Gauldie J. Fibrocytes and fibroblasts-where are we now. Int J Biochem Cell Biol (2019) 116:105595. doi: 10.1016/j.biocel.2019.105595 31473260

[25] Bianchetti L Barczyk M Cardoso J Schmidt M Bellini A Mattoli S. Extracellular matrix remodelling properties of human fibrocytes. J Cell Mol Med (2012) 16(3):483–95. doi: 10.1111/j.1582-4934.2011.01344.x PMC3822925 21595824

[26] Pilling D Fan T Huang D Kaul B Gomer RH. Identification of markers that distinguish monocyte-derived fibrocytes from monocytes, macrophages, and fibroblasts. PloS One (2009) 4(10):e7475. doi: 10.1371/journal.pone.0007475 19834619 PMC2759556

[27] Elhaj Mahmoud D Kaabachi W Sassi N Mokhtar A Ben Ammar L Rekik S. Expression of extracellular matrix components and cytokine receptors in human fibrocytes during rheumatoid arthritis. Connect Tissue Res (2021) 62(6):1–12. doi: 10.1080/03008207.2021.1873962 33427511

[28] Just SA Nielsen C Werlinrud JC Larsen PV Hejbøl EK Tenstad HB. Fibrocytes in early and long-standing rheumatoid arthritis: A 6-month trial with repeated synovial biopsy, imaging and lung function test. RMD Open (2021) 7(1):e001494. doi: 10.1136/rmdopen-2020-001494 33674419 PMC7938972

[29] Galligan CL Siminovitch KA Keystone EC Bykerk V Perez OD Fish EN. Fibrocyte activation in rheumatoid arthritis. Rheumatology (2010) 49(4):640–51. doi: 10.1093/rheumatology/kep265 PMC2909797 19858121

[30] Galligan CL Keystone EC Fish EN. Fibrocyte and T cell interactions promote disease pathogenesis in rheumatoid arthritis. J Autoimmun (2016) 69:38–50. doi: 10.1016/j.jaut.2016.02.008 26948996

[31] Miranda-Carús ME Balsa A Benito-Miguel M De Ayala CP Martín-Mola E. Rheumatoid arthritis synovial fluid fibroblasts express trail-R2 (Dr5) that is functionally active. Arthritis Rheum (2004) 50(9):2786–93. doi: 10.1002/art.20501 15457446

[32] Zafari P Rafiei A Faramarzi F Ghaffari S Amiri AH Taghadosi M. Human fibroblast-like synoviocyte isolation matter: A comparison between cell isolation from synovial tissue and synovial fluid from patients with rheumatoid arthritis. Rev Assoc Méd Bras (2021) 67:1654–8. doi: 10.1590/1806-9282.20210706 34909894

[33] Rosengren S Boyle DL Firestein GS. Acquisition, culture, and phenotyping of synovial fibroblasts. Methods Mol Med (2007) 135:365–75. doi: 10.1007/978-1-59745-401-8_24 17951672

[34] Mahmoud DE Kaabachi W Sassi N Mokhtar A Moalla M Ammar LB. Sfrp5 enhances Wnt5a induced-inflammation in rheumatoid arthritis fibroblast-like synoviocytes. Front Immunol (2021) 12:2356. doi: 10.3389/fimmu.2021.663683 PMC8239419 34211463

[35] Stephenson W Donlin LT Butler A Rozo C Bracken B Rashidfarrokhi A. Single-cell RNA-seq of rheumatoid arthritis synovial tissue using low-cost microfluidic instrumentation. Nat Commun (2018) 1:791. doi: 10.1038/s41467-017-02659-x PMC5824814 29476078

[36] Dho SH Lim JC Kim LK. Beyond the role of CD55 as a complement component. Immune Netw (2018) 1:e11. doi: 10.4110/in.2018.18.e11 PMC5833118 29503741

[37] De Sousa EB Casado PL Neto VM Duarte MEL. Aguiar DP.Synovial fluid and synovial membrane mesenchymal stem cells: latest discoveries and therapeutic perspectives. Stem Cell Res Ther (2014) 5:112. doi: 10.1186/scrt501 25688673 PMC4339206

[38] Karin N. CXCR3 ligands in cancer and autoimmunity, chemoattraction of effector T cells, and beyond. Front Immunol (2020) 11:976. doi: 10.3389/fimmu.2020.00976 32547545 PMC7274023

[39] De Bari C. Are mesenchymal stem cells in rheumatoid arthritis the good or bad guys? Arthritis Res Ther (2015) 1:113. doi: 10.1186/s13075-015-0634-1 PMC4416346 25929877

[40] Orr C Vieira-Sousa E Boyle DL Buch MH Buckley CD Cañete JD. Synovial tissue research: A state-of-the-Art review. Nat Rev Rheumatol (2017) 18:463–75. doi: 10.1038/nrrheum.2017.115 28701760

[41] Kemble S Croft AP. Critical role of synovial tissue–resident macrophage and fibroblast subsets in the persistence of joint inflammation. Front Immunol (2021) 12:715894. doi: 10.3389/fimmu.2021.715894 34539648 PMC8446662

[42] Chu CQ. Fibroblasts in rheumatoid arthritis. N Engl J Med (2020) 383:1679–81. doi: 10.1056/NEJMcibr2024718 33085868

[43] Zhang F Wei K Slowikowski K Fonseka CY Rao DA Kelly S. Defining inflammatory cell states in rheumatoid arthritis joint synovial tissues by integrating single-cell transcriptomics and mass cytometry. Nat Immunol (2019) 20(7):928–42. doi: 10.1038/s41590-019-0378-1 PMC6602051 31061532

[44] Friedenstein AJ Chailakhjan RK Lalykina KS. The development of fibroblast colonies in monolayer cultures of guinea-pig bone marrow and spleen cells. Cell Tissue Kinet (1970) 3:393–403. doi: 10.1111/j.1365-2184.1970.tb00347.x 5523063

[45] Caplan AI Bruder SP. Mesenchymal stem cells: building blocks for molecular medicine in the 21st century. Trends Mol Med (2001) 7:259–64. doi: 10.1016/s1471-4914(01)02016-0 11378515

[46] Dominici M Le Blanc K Mueller I Slaper-Cortenbach I Marini F Krause D. Minimal criteria for defining multipotent mesenchymal stromal cells. the international society for cellular therapy position statement. Cytotheraphy (2006) 8:315–17. doi: 10.1080/14653240600855905 16923606

[47] De Bari C Dell'Accio F Tylzanowski P Luyten FP. Multipotent mesenchymal stem cells from adult human synovial membrane. Arthritis Rheumatol (2001) 44:1928–42. doi: 10.1002/1529-0131(200108)44:8<1928::AID-ART331>3.0.CO;2-P 11508446

[48] Kohno Y Mizuno M Endo K Ozeki N Katano H Matsumoto M. Yields of mesenchymal stromal cells from synovial fluid reflect those from synovium in patients with rheumatoid arthritis. Tissue Cell (2022) 75:101727. doi: 10.1016/j.tice.2021.101727 34998163

[49] Park SJ Kim KJ Kim WU Cho CS. Interaction of mesenchymal stem cells with fibroblast-like synoviocytes *via* cadherin-11 promotes angiogenesis by enhanced secretion of placental growth factor. J Immunol (2014) 7:3003–10. doi: 10.4049/jimmunol.1302177 24574497

[50] Marinova-Mutafchieva L Williams RO Funa K Maini RN Zvaifler NJ. Inflammation is preceded by tumor necrosis factor-dependent infiltration of mesenchymal cells in experimental arthritis. Arthritis Rheum (2002) 2:507–13. doi: 10.1002/art.10126 11840454

[51] Nykanen P Helve T Kankaanpaa U Larsen A. Characterization of the DNA-synthesizing cells in rheumatoid synovial tissue. Scand J Rheumatol (1978) 7:118–22. doi: 10.3109/03009747809098848 81518

[52] Mousavi MJ Karami J Aslani S Tahmasebi MN Vaziri AS Jamshidi A. Transformation of fibroblast-like synoviocytes in rheumatoid arthritis; from a friend to foe. Auto Immun Highlight (2021) 12(1):1–13. doi: 10.1186/s13317-020-00145-x PMC7863458 33546769

[53] Ganesan R Rasool M. Fibroblast-like synoviocytes-dependent effector molecules as a critical mediator for rheumatoid arthritis: Current status and future directions. Int Rev Immunol (2017) 36(1):20–30. doi: 10.1080/08830185.2016.1269175 28102734

[54] Bottini N Firestein GS. Duality of fibroblast-like synoviocytes in Ra: Passive responders and imprinted aggressors. Nat Rev Rheumatol (2013) 9(1):24–33. doi: 10.1038/nrrheum.2012.190 23147896 PMC3970924

[55] Glossop JR Haworth KE Emes RD Nixon NB Packham JC Dawes PT. DNA Methylation profiling of synovial fluid fls in rheumatoid arthritis reveals changes common with tissue-derived fls. Epigenomics (2015) 7(4):539–51. doi: 10.2217/epi.15.15 26111028

[56] Baer C Claus R Frenzel LP Park YJ Gu L Weichenhan D. Extensive promoter DNA hypermethylation and hypomethylation is associated with aberrant microRNA expression in chronic lymphocytic leukemia. Cancer Res (2012) 15:3775–85. doi: 10.1158/0008-5472.CAN-12-0803 22710432

[57] Lee DM Kiener HP Agarwal SK Noss EH Watts GF Chisaka O. Cadherin-11 in synovial lining formation and pathology in arthritis. Science (2007) 315(5814):1006–10. 10.1126/science.1137306 17255475

[58] Kiener HP Lee DM Agarwal SK Brenner MB. Cadherin-11 induces rheumatoid arthritis fibroblast-like synoviocytes to form lining layers in vitro. Am J Pathol (2006) 168(5):1486–99. doi: 10.2353/ajpath.2006.050999 PMC1606584 16651616

[59] Kiener HP Niederreiter B Lee DM Jimenez-Boj E Smolen JS Brenner MB. Cadherin 11 promotes invasive behavior of fibroblast-like synoviocytes. Arthritis Rheum (2009) 60(5):1305–10. 10.1002/art.24453 PMC3764540 19404963

[60] Sen M Reifert J Lauterbach K Wolf V Rubin JS Corr M. Regulation of fibronectin and metalloproteinase expression by wnt signaling in rheumatoid arthritis synoviocytes. Arthritis Rheum (2002) 46(11):2867–77. doi: 10.1002/art.10593 12428226

[61] Carson S Lavietes B Diamond H Kiney S. The immunoreactivity, ligand, and cell binding characteristics of rheumatoid synovial fluid flbronectin. Arthritis Rheum (1985) 28(60):1–612. doi: 10.1002/art.1780280602 4004971

[62] Sen M. Wnt signalling in rheumatoid arthritis. Rheumatology (2005) 44(6):708–13. doi: 10.1093/rheumatology/keh553 15705634

[63] Rodriguez-Trillo A Mosquera N Pena C Rivas-Tobío F Mera-Varela A Gonzalez A. Non-canonical Wnt5a signaling through ryk contributes to aggressive phenotype of the rheumatoid fibroblast-like synoviocytes. Front Immunol (2020) 11:555245 . 33178184 10.3389/fimmu.2020.555245 PMC7593687

[64] Tolboom T Pieterman E van der Laan W Toes R Huidekoper A Nelissen R. Invasive properties of fibroblast-like synoviocytes: Correlation with growth characteristics and expression of mmp-1, mmp-3, and mmp-10. Ann Rheum Dis (2002) 61(11):975–80. doi: 10.1136/ard.61.11.975 PMC1753950 12379519

[65] Nagase H Visse R Murphy G. Structure and function of matrix metalloproteinases and timps. Cardiovasc Res (2006) 69(3):562–73. doi: 10.1016/j.cardiores.2005.12.002 16405877

[66] Morrison CJ Butler GS Rodríguez D Overall CM. Matrix metalloproteinase proteomics: substrates, targets, and therapy. Curr Opin Cell Biol (2009) 21(5):645–53. doi: 10.1016/j.ceb.2009.06.006 19616423

[67] Nagase H Woessner JF. Matrix metalloproteinases. J Biol Chem (1999) 274(31):21491–4. doi: 10.1074/jbc.274.31.21491 10419448

[68] Yoshihara Y Yamada H. Matrix metalloproteinases and cartilage matrix degradation in rheumatoid arthritis. Clin calcium (2007) 17(4):500–8. 17404478

[69] Zhao W Zhang C Shi M Zhang J Li M Xue X. The discoidin domain receptor 2/Annexin A2/Matrix metalloproteinase 13 loop promotes joint destruction in arthritis through promoting migration and invasion of fibroblast-like synoviocytes. Arthritis Rheum (2014) 66(9):2355–67. doi: 10.1002/art.38696 24819400

[70] Herzog EL Bucala R. Fibrocytes in health and disease. Exp Hematol (2010) 38(7):548–56. doi: 10.1016/j.exphem.2010.03.004 PMC3136351 20303382

[71] Svensson MND Zoccheddu M Yang S Nygaard G Secchi C Doody KM. Synoviocyte-targeted therapy synergizes with TNF inhibition in arthritis reversal. Sci Adv (2020) 26:eaba4353. doi: 10.1126/sciadv.aba4353 PMC7319753 32637608

[72] Finch R Sostelly A Sue-Ling K Blaeuer A Duchateau-Nguyen Lidia Ukarma G. op0224 results of a phase 2 study of rg6125, an anti-cadherin-11 monoclonal antibody, in rheumatoid arthritis patients with an inadequate response to anti-tnfalpha therapy. BMJ (2019) 78(2):189. doi: 10.1136/annrheumdis-2019-eular.3028

[73] Wei K Korsunsky I Marshall JL Gao A Watts GFM Major T. Notch signaling drives synovial fibroblast identity and arthritis pathology. Nature (2020) 582:259–64. doi: 10.1038/s41586-020-2222-z PMC7841716 32499639

